# Estimation of treatment effects in observational stroke care data: comparison of statistical approaches

**DOI:** 10.1186/s12874-022-01590-0

**Published:** 2022-04-10

**Authors:** Marzyeh Amini, Nikki van Leeuwen, Frank Eijkenaar, Rob van de Graaf, Noor Samuels, Robert van Oostenbrugge, Ido R. van den Wijngaard, Pieter Jan van Doormaal, Yvo B. W. E. M. Roos, Charles Majoie, Bob Roozenbeek, Diederik Dippel, James Burke, Hester F. Lingsma, on behalf of the, MR CLEAN Registry Investigators, Diederik W. J. Dippel, Aad van der Lugt, Charles B. L. M. Majoie, Yvo B. W. E. M. Roos, Robert J. van Oostenbrugge, Wim H. van Zwam, Jelis Boiten, Jan Albert Vos, Josje Brouwer, Sanne J. den Hartog, Wouter H. Hinsenveld, Manon Kappelhof, Kars C. J. Compagne, Robert-Jan B. Goldhoorn, Maxim J. H. L. Mulder, Ivo G. H. Jansen, Diederik W. J. Dippel, Bob Roozenbeek, Aad van der Lugt, Adriaan C. G. M. van Es, Charles B. L. M. Majoie, Yvo B. W. E. M. Roos, Bart J. Emmer, Jonathan M. Coutinho, Wouter J. Schonewille, Jan Albert Vos, Marieke J. H. Wermer, Marianne A. A. van Walderveen, Julie Staals, Robert J. van Oostenbrugge, Wim H. van Zwam, Jeannette Hofmeijer, Jasper M. Martens, Geert J. Lycklama à Nijeholt, Jelis Boiten, Sebastiaan F. de Bruijn, Lukas C. van Dijk, H. Bart van der Worp, Rob H. Lo, Ewoud J. van Dijk, Hieronymus D. Boogaarts, J. de Vries, Paul L. M. de Kort, Julia van Tuijl, Jo Jo P. Peluso, Puck Fransen, Jan S. P. van den Berg, Boudewijn A. A. M. van Hasselt, Leo A. M. Aerden, René J. Dallinga, Maarten Uyttenboogaart, Omid Eschgi, Reinoud P. H. Bokkers, Tobien H. C. M. L. Schreuder, Roel J. J. Heijboer, Koos Keizer, Lonneke S. F. Yo, Heleen M. den Hertog, Emiel J. C. Sturm, Paul Brouwers, Charles B. L. M. Majoie, Wim H. van Zwam, Aad van der Lugt, Geert J. Lycklama à Nijeholt, Marianne A. A. van Walderveen, Marieke E. S. Sprengers, Sjoerd F. M. Jenniskens, René van den Berg, Albert J. Yoo, Ludo F. M. Beenen, Alida A. Postma, Stefan D. Roosendaal, Bas F. W. van der Kallen, Ido R. van den Wijngaard, Adriaan C. G. M. van Es, Bart J. Emmer, Jasper M. Martens, Lonneke S. F. Yo, Jan Albert Vos, Joost Bot, Pieter-Jan van Doormaal, Anton Meijer, Elyas Ghariq, Reinoud P. H. Bokkers, Marc P. van Proosdij, G. Menno Krietemeijer, Jo P. Peluso, Hieronymus D. Boogaarts, Rob Lo, Dick Gerrits, Wouter Dinkelaar, Auke P. A. Appelman, Bas Hammer, Sjoert Pegge, Anouk van der Hoorn, Saman Vinke, Diederik W. J. Dippel, Aad van der Lugt, Charles B. L. M. Majoie, Yvo B. W. E. M. Roos, Robert J. van Oostenbrugge, Wim H. van Zwam, Geert J. Lycklama à Nijeholt, Jelis Boiten, Jan Albert Vos, Wouter J. Schonewille, Jeannette Hofmeijer, Jasper M. Martens, H. Bart van der Worp, Rob H. Lo, Robert J. van Oostenbrugge, Jeannette Hofmeijer, H. Zwenneke Flach, Hester F. Lingsma, Naziha el Ghannouti, Martin Sterrenberg, Corina Puppels, Wilma Pellikaan, Rita Sprengers, Marjan Elfrink, Michelle Simons, Marjolein Vossers, Joke de Meris, Tamara Vermeulen, Annet Geerlings, Gina van Vemde, Tiny Simons, Cathelijn van Rijswijk, Gert Messchendorp, Nynke Nicolaij, Hester Bongenaar, Karin Bodde, Sandra Kleijn, Jasmijn Lodico, Hanneke Droste, Maureen Wollaert, Sabrina Verheesen, D. Jeurrissen, Erna Bos, Yvonne Drabbe, Michelle Sandiman, Marjan Elfrink, Nicoline Aaldering, Berber Zweedijk, Mostafa Khalilzada, Jocova Vervoort, Hanneke Droste, Nynke Nicolaij, Michelle Simons, Eva Ponjee, Sharon Romviel, Karin Kanselaar, Erna Bos, Denn Barning, Esmee Venema, Vicky Chalos, Ralph R. Geuskens, Tim van Straaten, Saliha Ergezen, Roger R. M. Harmsma, Daan Muijres, Anouk de Jong, Olvert A. Berkhemer, Anna M. M. Boers, J. Huguet, P. F. C. Groot, Marieke A. Mens, Katinka R. van Kranendonk, Kilian M. Treurniet, Ivo G. H. Jansen, Manon L. Tolhuisen, Heitor Alves, Annick J. Weterings, Eleonora L. F. Kirkels, Eva J. H. F. Voogd, Lieve M. Schupp, Sabine Collette, Adrien E. D. Groot, Natalie E. LeCouffe, Praneeta R. Konduri, Haryadi Prasetya, Nerea Arrarte-Terreros, Lucas A. Ramos

**Affiliations:** 1 Department of Public Health, Erasmus University Medical Center, Erasmus MC, P.O. Box 2040, CA Rotterdam, the Netherlands; 2 Erasmus School of Health Policy & Management, Erasmus University Rotterdam, Rotterdam, the Netherlands; 3 Department of Radiology and Nuclear Medicine, Erasmus University Medical Center, Rotterdam, the Netherlands; 4 Department of Neurology, Erasmus University Medical Center, Rotterdam, the Netherlands; 5 Maastricht University Medical Center, Maastricht, the Netherlands; 6 Department of Neurology, Haaglanden Medical Center, the Hague, the Netherlands; 7 Amsterdam University Medical Centers, location AMC, Amsterdam, the Netherlands; 8 Department of Radiology and Nuclear Medicine, Amsterdam University Medical Centers, location AMC, Amsterdam, the Netherlands; 9 Department of Neurology, University of Michigan, Ann Arbor, MI USA

**Keywords:** Unmeasured confounding, Confounding by indication, Statistical approaches, Ecological-analysis, Instrumental variable, Acute ischemic stroke, Intravenous thrombolysis, General anesthesia

## Abstract

**Introduction:**

Various statistical approaches can be used to deal with unmeasured confounding when estimating treatment effects in observational studies, each with its own pros and cons. This study aimed to compare treatment effects as estimated by different statistical approaches for two interventions in observational stroke care data.

**Patients and methods:**

We used prospectively collected data from the MR CLEAN registry including all patients (*n* = 3279) with ischemic stroke who underwent endovascular treatment (EVT) from 2014 to 2017 in 17 Dutch hospitals. Treatment effects of two interventions – i.e., receiving an intravenous thrombolytic (IVT) and undergoing general anesthesia (GA) before EVT – on good functional outcome (modified Rankin Scale ≤2) were estimated. We used three statistical regression-based approaches that vary in assumptions regarding the source of unmeasured confounding: individual-level (two subtypes), ecological, and instrumental variable analyses. In the latter, the preference for using the interventions in each hospital was used as an instrument.

**Results:**

Use of IVT (range 66–87%) and GA (range 0–93%) varied substantially between hospitals. For IVT, the individual-level (OR ~ 1.33) resulted in significant positive effect estimates whereas in instrumental variable analysis no significant treatment effect was found (OR 1.11; 95% CI 0.58–1.56). The ecological analysis indicated no statistically significant different likelihood (β = − 0.002%; *P* = 0.99) of good functional outcome at hospitals using IVT 1% more frequently. For GA, we found non-significant opposite directions of points estimates the treatment effect in the individual-level (ORs ~ 0.60) versus the instrumental variable approach (OR = 1.04). The ecological analysis also resulted in a non-significant negative association (0.03% lower probability).

**Discussion and conclusion:**

Both magnitude and direction of the estimated treatment effects for both interventions depend strongly on the statistical approach and thus on the source of (unmeasured) confounding. These issues should be understood concerning the specific characteristics of data, before applying an approach and interpreting the results. Instrumental variable analysis might be considered when unobserved confounding and practice variation is expected in observational multicenter studies.

**Supplementary Information:**

The online version contains supplementary material available at 10.1186/s12874-022-01590-0.

## Introduction

Assignment of treatment mostly depends on both measured and unmeasured patient characteristics (i.e., prognostic factors) that are associated with outcome [1, 2]. As a consequence, estimates of treatment effects in observational data tend to be biased because they reflect, at least in part, the effects of (unmeasured) confounding variables rather than the true effect of treatment [1, 2]. In randomized clinical trials (RCTs), such (unmeasured) confounding is avoided because randomization ensures all prognostic factors to be equally balanced between treatment groups [3]. However, RCTs can be expensive and challenging to conduct. In addition, for ethical and practical reasons executing an RCT could even be infeasible, or it could provide results with considerable delay. Furthermore, the external validity of RCTs might be limited due to often stringent inclusion criteria [2–4].

In practice, therefore, observational studies constitute the main alternative in absence of RCTs to approximate the true treatment effects of medical interventions. Several approaches – e.g. covariate adjustment, ecological analysis, and instrumental variable analysis – have been proposed to detect or control for (un) measured confounding in such studies [4–10]. With covariate adjustment, it is possible to directly adjust the treatment effect estimate for differences in measured prognostic factors in patients (e.g., disease severity) as well as in hospital factors (e.g., admission characteristics or practice style) that may influence the outcome [5]. An important downside of this approach is that it does not allow for controlling for differences of unmeasured factors that may influence both treatment allocation and outcome, causing confounding by indication. Moreover, sufficient sample sizes are required to allow for adequate statistical modeling of multiple confounding variables [5]. The ecological analysis approach is based on the analysis of differences in treatment decisions between groups of patients, i.e., at the hospital-level [4, 7, 8, 11]. Although ecological studies may reduce the effect of unmeasured confounding, this approach introduces new problems that are not present in individual-level analyses, such as the ecological fallacy, collinearity, and lack of power [11]. A third approach is instrumental variable analysis [6, 8]. This approach takes advantage of both reduced confounding by indication in the ecological analysis and more accurate specification of individual outcome and potential confounders using the individual patient data (just like when applying covariate adjustment). With instrumental variable analysis, a measured variable (i.e., the instrument) is used that influences treatment decisions but that is assumed to be unrelated to (both measured and unmeasured) patient characteristics that influence the outcome. When substantial hospital-level variation in the use of a specific intervention exists, the variable ‘hospital’ can be used as an instrument to study the effect of this intervention on the outcome [6, 10]. Choosing an appropriate instrument is the main challenge when using this approach.

In stroke care, endovascular treatment (EVT) has become standard care for patients with acute ischemic stroke caused by an intracranial large vessel occlusion of the anterior circulation, when the procedure is available and can be performed in a timely fashion [12–15]. All major guidelines recommend intravenous thrombolysis (IVT) in eligible patients before EVT [16, 17]. Patients receive IVT with alteplase as standard care, unless they have a contraindication for IVT. In addition, it might be necessary to perform the EVT procedure under a form of anesthesia, like conscious sedation or general anesthesia (GA), in case the patient is agitated, or a secured airway is required [18]. The effect of IVT and GA on neurological outcomes after EVT were evaluated in previous RCTs [18–25] and observational studies [16, 18, 26], but the results were inconclusive.

Given the large between-hospital variability in IVT and GA utilization before EVT [27], this study aimed to compare treatment effects of both IVT and GA estimated using observational data derived from a nationwide stroke care registry, to provide further insight into the influence of different analysis approaches and source of (un) measured confounding.

## Methods

### Study design and patients

For the current study, we used data collected between March 2014 and November 2017 from the MR CLEAN Registry, a prospective, observational study in all 17 hospitals that perform EVT in the Netherlands [28]. We included patients adhering to the following criteria: treatment at age 18 years and older, treatment in a hospital that participated in the MR CLEAN trial, clinical diagnosis of acute stroke with a deficit on the National Institute of Health Stroke Scale (NIHSS) of at least 2 points, CT or MRI ruling out intracranial hemorrhage, the possibility to start treatment within 6 h of onset, and proximal intracranial vessel occlusion in the anterior circulation (internal carotid artery, internal carotid artery terminus, middle (M1/M2) cerebral artery, or anterior (A1/A2) cerebral artery), as shown by computed tomography angiography. Details on the study design and objectives of the MR CLEAN Registry have been described elsewhere [28]. Overall, data from 3279 patients were available for analysis.

### Variables

#### Outcome measure

Good functional outcome as measured by a score of 0–2 on the modified Rankin Scale (mRS) was used as the outcome [29]. The mRS is a commonly used measure of patients’ functional outcome after ischemic stroke care and ranges from 0 (no symptoms) to 6 (death). The mRS score was assessed at 90 days after EVT (± 14 days).

#### Treatment variables

We assessed two treatment variables. First, yes/no IVT administration before EVT was studied. Second, yes/no GA during EVT was assessed, no GA meaning either no sedation or conscious sedation.

#### Case-mix variables

Time between stroke onset and arrival at the emergency department (ED) of the EVT hospital and patients’ age, sex, relevant medical history (i.e. previous stroke, atrial fibrillation, hypertension, hypercholesterolemia), and baseline score on the NIHSS were considered potential confounders of the estimated association between administration of IVT or GA and good functional outcome. The latter four variables were selected based on clinical knowledge and previous studies [30, 31]. The time between stroke onset and arrival at the ED was used because it cannot be influenced by hospitals while it may have an impact on the outcome.

### Statistical analysis

All analyses were carried out using SPSS version 25.0 (IBM Corporation, Armonk, NY, USA) and Stata version 13.0 (StataCorp, College Station, TX, USA). Case-mix variables and outcome were described using summary statistics and measures of spread, and differences therein among hospitals and patient groups (i.e., yes/no IVT and GA) were tested on statistical significance (*P* < 0.05). Groups were compared using a non-parametric Kruskal Wallis test for continuous variables or Pearson’s chi-square statistic for categorical variables. Next, separately for IVT and GA, we applied three statistical approaches to estimate and compare treatment effects, both unadjusted and case-mix adjusted: individual-level, ecological, and instrumental variable analysis.

#### Individual-level analysis

For the individual-level analysis we ran two separate models for each of the two interventions, with good functional outcome after 90 days (i.e., mRS 0–2) as the dependent variable: standard logistic regression and generalized estimating equations (GEE).

Logistic regression assumes independent observations for every patient. But in multicenter studies, it is not necessarily valid when individual observations (here patients) and their characteristics are ‘clustered’ in higher-level entities (here hospitals) [32]. Therefore, given differences among hospitals in the way they treat patients, correlation is likely within-hospital observations. The GEE model allows for accounting for this correlation (differences in the use of IVT or GA between hospitals) to appropriately adjust the estimation of effect sizes and confidence intervals [32]. In the case of significant within-hospital correlation, broader confidence intervals are estimated due to fewer independent observations. We accounted for clustering by hospitals using a compound symmetry correlation structure (correlation assumed to be the same for all within-hospital comparisons and all between-hospital comparisons). This means that we assumed the treatment effects were the same within the same cluster (hospital), and there was no variance between patients. Given our binary outcome variable, we used a binomial distribution and logistic link function for the GEE model [33].

#### Ecological analysis

Ecological analysis exploits differences in preferences for the use of IVT or GA between groups of patients (i.e., hospitals). The key assumption is that differences in the use of interventions across hospitals are mainly driven by hospitals’ practice styles rather than differences in patients’ prognostic factors. While practice style is difficult to detect at the individual patient level, its impact can be measured ecologically. Linear regression at the hospital-level was used to estimate the association between the percentage of patients with a good functional outcome (dependent variable) and the percentage of patients who received IVT or GA. To let high-volume hospitals contribute more to the analysis than low-volume hospitals, we used the number of patients treated in each hospital as weights. In the adjusted analysis, case-mix variables were measured at the hospital-level (i.e., mean age, the proportion of patients with the male sex, proportion of patients with a previous stroke, atrial fibrillation, hypertension, and hypercholesterolemia, mean NIHSS score, and mean time from onset to ED-arrival). This analysis provides insight into the change in absolute probability of a good functional outcome for every additional 1% of patients receiving IVT or GA.

#### Instrumental variable analysis

**Fig. 1:**
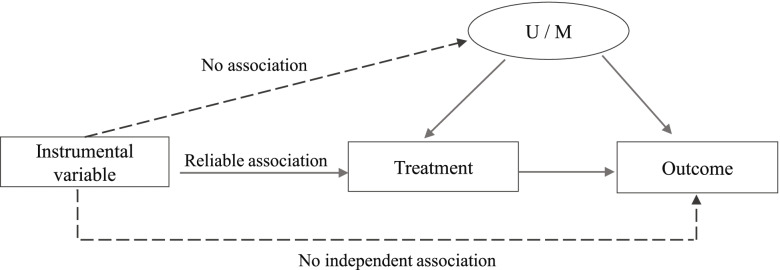
Instrumental variable assumptions in relation to estimation of treatment effect on the outcome. U/M Unmeasured/measured confounders

The advantages of considering the hospital’s practice style in the ecological analysis and the potentially more accurate specification of individual-level outcome and potential confounders in the individual-level analysis, were combined in the instrumental variable analysis. Specifically, preference for IVT or GA per hospital was used as an instrument, defined as the proportion of patients who received IVT or GA within each hospital [34, 35]. By using this instrument, we relied on three key assumptions for instrumental variable analysis (Fig. 1): (1) the instrument is associated with exposure to the treatment; (2) the instrument only has an effect on the outcome *through* treatment; and [3] the instrument is unrelated to (un) measured prognostic factors. Using the Stata command “*ivregress gmm*” [36] we used two-stage logistic regression to estimate the treatment effect on good functional outcome (Supplementary information). The first stage comprised running a case-mix adjusted logistic regression model to estimate the predicted probability of receiving IVT or GA given the preference to use those interventions at that hospital. In the second stage, the predicted probability of the first stage was used as a covariate in another logistic regression model at the individual patient level adjusted for case-mix variables, with yes/no good functional outcome as a dependent variable. In addition, the cluster effect of hospitals was added to the analysis and the standard errors were adjusted for 17 clusters. This model yields an estimate of the effect of IVT or GA on good functional outcome. Then, we assessed the validity of the instrumental variable analysis. Specifically, based on the first abovementioned assumption, the strength of the instrument was assessed using the *F*-statistic calculated as [*R*^2^ × (n − 1 − K)]/[(1 − *R*^2^) × K], where ‘*R*^2^’ represents the partial variance from the first-stage, ‘n’ represents the sample size, and ‘K’ represents the number of instrumental variables included in the model [36, 37]. As a rule of thumb, the *F*-statistic for the significance of the instrument in the first stage should exceed 10. The second assumption was tested using the Durbin-Wu-Hausman χ^2^ test to examine the existence of endogeneity in the estimated treatment effects. This test compares an estimate of the average treatment effect assuming no unmeasured confounding, to an estimate of the average treatment effect using an instrument that allows for unmeasured confounding [38]. The third assumption underlying instrumental variable analysis we tested was whether the instrumental variable was associated with case-mix variables.

## Results

### Descriptive analyses

**Fig. 2:**
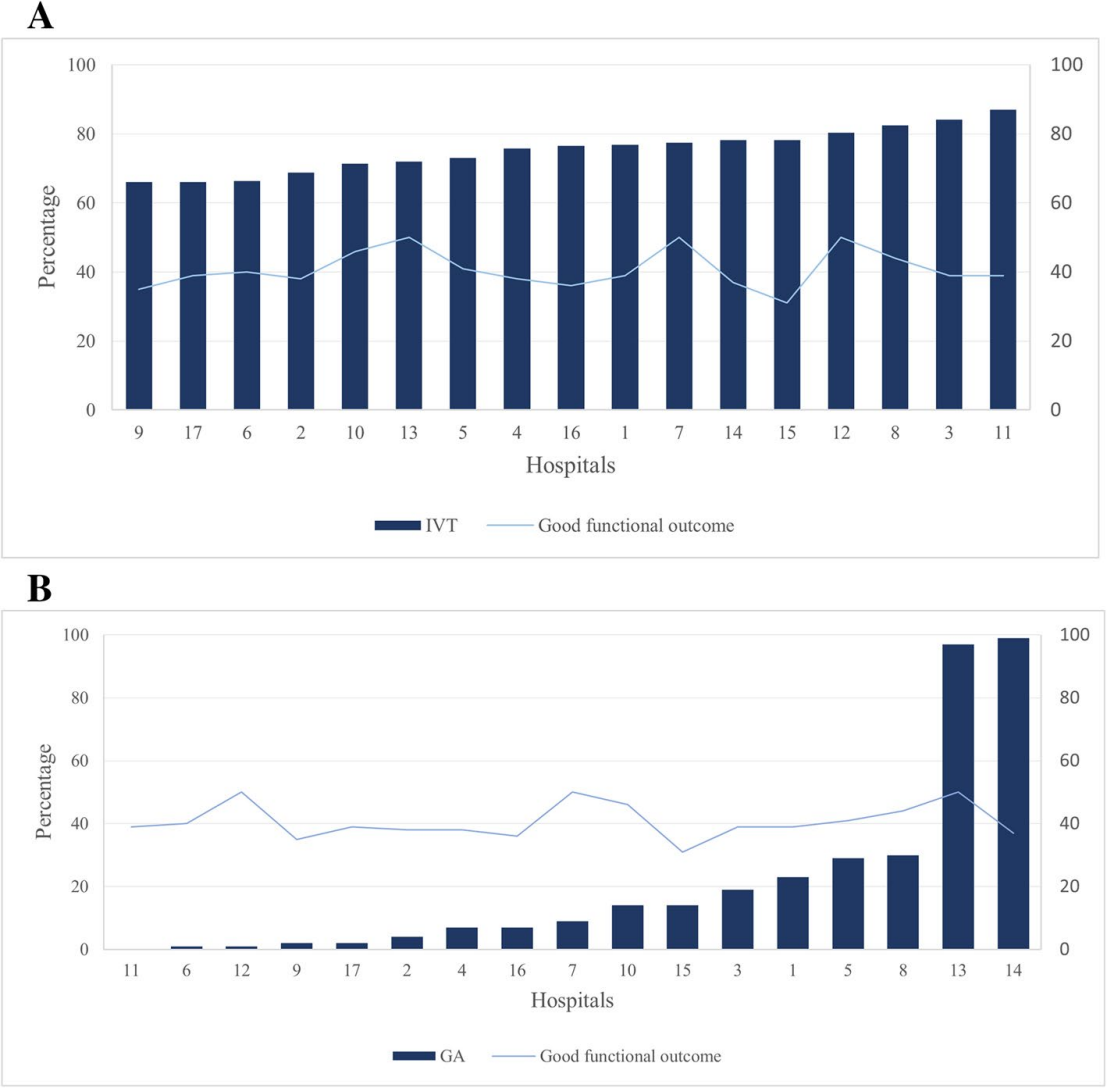
Differences in the percentage of patients receiving IVT (**A**) and undergoing general anesthesia (**B**) before EVT and good functional outcome in 17 EVT hospitals in the Netherlands. *Note*: Good functional outcome is defined as mRS 0–2 at 90 days

The number of patients receiving IVT varied significantly between hospitals (range 66–87%, *P* < 0.01) and GA (range 0–93%, *P* < 0.01) (Fig. 2). The number of EVT patients also varied widely from 23 to 405 patients per hospital (Table 1). There were significant differences (*P* < 0.05) in case-mix variables between patients who received the interventions of interest (i.e., IVT and GA) and patients who did not (Table 1).

**Table 1:** Case-mix characteristics of patients treated in all 17 EVT hospitals in the Netherlands; stratified by whether patients received IVT or not and whether patients underwent GA or not

	hospitals range (*n* = 17)	*P*-values^b^	IVT + (*n* = 2450)	IVT - (*n* = 817)	*P*-values^b^	GA + (*n* = 778)	GA - (*n* = 2304)	*P*-values^c^
Age (years) [Median (IQR)]	68–77	0.001	71 (60–80)	73 (63–82)	< 0.001	70 (59–79)	73 (62–81)	< 0.001
Baseline NIHSS score [Median (IQR)]	13–17	< 0.001	16 (11–19)	16 (11–20)	0.048	17 (12–20)	16 (11–19)	0.011
Time from onset to arrival at the ED (min) [Median (IQR)]	52–160	< 0.001	135 (65–185)	145 (67–240)	< 0.001	140 (72–190)	135 (64–195)	0.538
Men [N (%)]	39–55%	0.790	1294 (53)	394 (48)	0.024	420 (54)	1178 (51)	0.171
Medical History [N (%)]								
Previous Stroke	0–26%	< 0.001	330 (14)	212 (26)	< 0.001	127 (16)	389 (17)	0.739
Atrial Fibrillation	13–37%	< 0.001	414 (17)	356 (44)	< 0.001	157 (20)	567 (25)	0.012
Hypertension	41–67%	< 0.001	1234 (50)	447 (55)	0.037	346 (45)	1251 (54)	< 0.001
Hypercholesterolemia	15–50%	< 0.001	696 (28)	269 (33)	0.023	207 (27)	720 (31)	< 0.001
Good functional outcome^a^ [N (%)]	31–50%	0.004	985 (40)	251 (30)	< 0.001	272 (35)	896 (39)	0.309
IVT utilization [N (%)]	66–87%	< 0.001						
GA utilization [N (%)]	0–99%	< 0.001						
Hospital volume [n]	23–405							

*IVT* Intravenous thrombolysis, *GA* general anesthesia, *NIHSS* National institutes of health stroke scale, *ED* Emergency department

^a^ Good functional outcome is defined as mRS 0–2 at 90 days

^b^ *P-value* is based on comparison between 17 centers using a non-parametric Kruskal Wallis test for continuous variables or Pearson’s chi-square statistic for categorical variables

^c^ *P-value* is based on comparison between groups (receiving IVT/GA vs. non-receiving IVT/GA) using a non-parametric Kruskal Wallis test for continuous variables or Pearson’s chi-square statistic for categorical variables

### IVT intervention

**Fig. 3:**
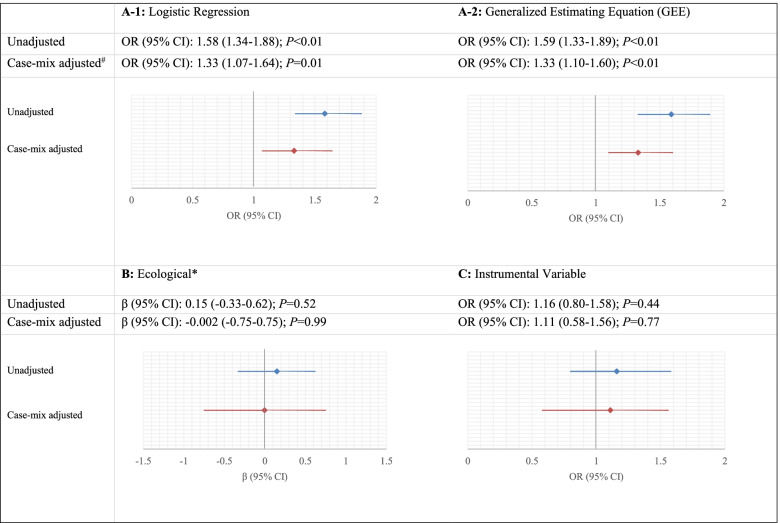
Effect estimates of receiving IVT intervention on the good functional outcome (mRS 0–2 at 90 days) from four statistical methods of **A-1** logistic regression, **A-2** generalized estimating equation, **B** ecological analysis, and **C** instrumental variable analysis. ^#^ Case-mix variables in the models are including age, sex, medical history, NIHSS score baseline, and time from onset to arrival at the ED of intervention hospital. Hospital volume was also added to the instrumental variable analysis. * Difference in the absolute probability of a good functional outcome for every 1% of the cases receiving IVT before EVT. For example, the unadjusted coefficient implies that the absolute probability of a good functional outcome is 0.15% higher for a patient treated at a hospital utilizing IVT intervention in 1% of the cases compared with one not utilizing the intervention

In the adjusted logistic regression model, receiving IVT was significantly associated with higher odds of good functional outcome (Fig. 3A-1). As the within-hospital correlation between patients was very small (0.0053), the estimated odds ratios and width of the confidence intervals in the GEE model were very similar to those in the logistic regression model (Fig. 3A-2). The same pattern was noticed in the unadjusted models, in which larger treatment effect estimates were estimated, as compared to the adjusted models (Figs. 3A-1 and A-2).

In the ecological analysis at the hospital-level, no statistically significant different likelihood (β = − 0.002%; *P* = 0.99) of good functional outcome at hospitals using IVT 1% more frequently was estimated after case-mix adjustment (Fig. 3B). In the case-mix adjusted instrumental variable analysis, no significant treatment effect was found when patients treated in hospitals were using IVT more frequently (OR 1.11; 95% CI 0.58–1.56; Fig. 3C). These results were not significantly different from the individual-level estimates from the logistic and GEE analyses (Durbin-Wu-Hausman χ^2^ = 0.073, *P-value* for difference = 0.75), and no endogeneity was noticed. The instrument explained 1.6% of the variation in the use of IVT in the adjusted model. The derived *F*-statistics was 41.2 in the adjusted model, which indicates that the instrument has sufficient power. However, we did find the instrument to be associated with some confounders including age, previous stroke, previous atrial fibrillation, and baseline NIHSS score (Supplementary Table 1). Thus, the assumptions underlying an appropriate instrumental variable analysis were not fully met.

### GA intervention

**Fig. 4:**
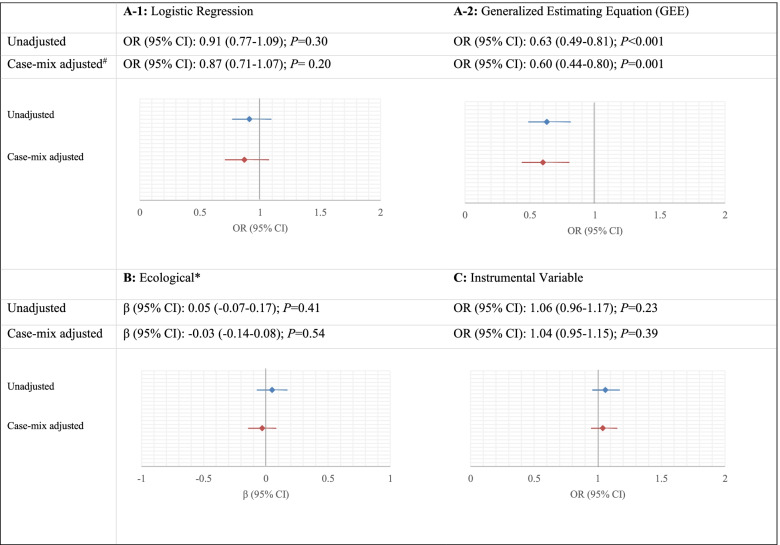
Effect estimates of undergoing general anesthesia on the good functional outcome (mRS 0–2 at 90 days) from four statistical methods of **A-1** logistic regression, **A-2** generalized estimating equation, **B** ecological analysis, and **C** instrumental variable analysis. ^#^ Case-mix variables in the models are including age, sex, medical history, NIHSS score baseline, and time from onset to arrival at the ED of intervention hospital. Hospital volume was also added to the instrumental variable analysis. * Difference in the absolute probability of a good functional outcome for every 1% of the cases receiving general anesthesia. For example, the unadjusted coefficient implies that the absolute probability of a good functional outcome is 0.05% higher for a patient treated at a hospital utilizing general anesthesia in 1% of the cases compared with one not utilizing the intervention

The adjusted logistic regression analyses of patients undergoing GA on good functional outcome showed no statistically significant effect (OR 0.87; 95% CI 0.71–1.07; Fig. 4A-1). The within-hospital correlation was 0.022 and in the adjusted GEE model the treatment effect was statistically significant (OR 0.60; 95% CI 0.44–0.80; Fig. 4A-2) and larger compared to the effect in the logistic regression model.

The ecological analysis showed no statistically significant different likelihood of a good functional outcome in the adjusted model, following a 1% increase in the use of GA (Fig. 4B).

In contrast to the individual-level results, the instrumental variable analysis showed that the use of GA has a positive but non-significant effect on a good functional outcome (OR 1.04; 95% CI 0.95–1.15; Fig. 4C). Durbin-Wu-Hausman χ^2^ was significant (17.6, *P* < 0.01) in the adjusted model, which indicates that one of the assumptions underlying the instrumental variable is met. The instrumental variable explained about 5.9% of the variation in the use of GA, regardless of case-mix adjustment. The *F*-statistic was 11,243.5, indicating a sufficiently powerful instrument. In addition, associations between the instruments and measured confounders were generally small (Supplementary Table 1), which indicates that another assumption underlying the instrumental variable was met.

## Discussion

The challenge when using observational data to estimate treatment effects is to overcome unmeasured confounding [8, 39, 40]. In the current study, we applied and compared various analytical approaches that have been advocated to reduce confounding in observational data, to assess the effect of IVT and GA on the probability of good functional outcome after ischemic stroke due to a large vessel occlusion in patients treated with EVT. We found that the size and direction of estimated effects strongly depend on the statistical approach used and therefore on the source of unmeasured confounding, i.e. individual patient prognostic factor or hospital practice.

In observational studies, regression analysis adjusted for patient characteristics is commonly used to estimate treatment effects of interventions. Our results of these analyses showed that patients who received IVT had significantly better outcomes than patients who did not receive IVT. This was consistent with the recent RCTs [22, 41] and inconsistent with other trials [18, 24, 25] results. Our adjusted regression analysis showed that patients receiving GA had no statistically significant higher odds of good functional outcome. Previous RCTs showed no conclusive results on the effect of GA versus other types of anesthesia [20, 21, 23]. In those trials, the comparator was different than the comparator in our study. In our data ‘no GA’ included patients with either no sedation or conscious sedation, but in those trials, GA was compared with conscious sedation only or local anesthesia. Generally speaking, individual-level regression analysis could provide unbiased estimates of treatment effects if all relevant confounders are known, measured, and adjusted for [5, 6]. But because of the difficulty to assess medical indications and underlying disease severity and prognosis, confounding by indication is often an impassable problem in multivariable regression analysis at the individual-level [5, 6]. In our observational data, some important prognostic variables were available (e.g., age, comorbidity, the severity of disease) and treatment groups were unbalanced concerning these variables. Still, it is possible that we have missed other important unmeasured prognostic factors, which might be associated with outcome. For example, many patients received IVT in a primary stroke center and were transferred to an intervention center. These patients were treated with EVT following no response to IVT. Also, many patients in the dataset were not eligible for IVT since they were outside the time window for treatment or because of other contraindications. Therefore, the treatments effects of IVT or GA as estimated in our individual-level analyses may be biased. This also could explain the different effect estimates in our analyses compared to the results in the RCTs. Additionally, an alternative approach for the measured confounding factor adjustment is the propensity score statistical methods, such as propensity score matching or inverse probability of treatment weighting, that may put different results [42, 43].

In addition, in multicenter studies at the individual-level, treatment effects are likely to be biased when observations are not independent but clustered at the hospital-level. To deal with this and to adjust for the potential effects of unmeasured hospital characteristics, GEE can be used [44]. For the IVT intervention, the within-hospital correlation between patients was very small and thus the GEE and logistic regression model yielded very similar results. In contrast, for GA the within-hospital correlation was moderate, resulting in no statistically significant effect in the logistic regression model compared to a significantly lower odds of good functional outcome in the GEE model. Similar to GEE, other approaches like random effect analysis which deal with dependent observations can put a similar argument (Supplementary Fig. 1 and Supplementary Fig. 2). In the presence of hospital-level clustering and unmeasured hospital characteristics influencing the outcome, GEE or other approaches which deal with clustering will be superior to conventional regression analysis for treatment effect estimation. However, this type of analysis might still be biased in the case of a small number of hospitals and/or small within-hospital sample size [44].

In an ecological analysis, aggregated data instead of individual-level data is used. Only the proportion of patients who received the intervention at a given hospital and aggregated information on case-mix was included. This approach is adjusted for confounding by hospital practice, especially in the case of a large variation in the use of the intervention between hospitals (as in the current study) [7]. For both interventions, observed differences in treatment effect estimates in the hospital-level versus the individual-level analyses suggest unmeasured confounding by hospital practice to be present to some extent. An ecological analysis at the level of the hospital bypasses the issue of confounding by indication. Patients’ prognostic factors are no longer placed in the model—only the aggregate values for a hospital’s treated population are considered. The portion of cases treated with interventions at a given hospital is more likely to be influenced by practice style rather than the prognostic factors of the case mix, particularly because some hospitals do not utilize the interventions. Yet, given its hospital-level approach, ecological analysis is prone to bias. For example, some hospitals treated more patients who were transferred from other hospitals, suggesting differences in some patient characteristics, which may have resulted in a change in the probability of a good functional outcome. The intuitive solution of adding these factors to the model (if available) would weaken rather than strengthen the analysis. In our analysis, the model had just 17 observations and already 8 independent variables, and adding additional variables would (further) increase the potential for collinearity and reduce power [11]. Another potential issue is the ecological fallacy [11], which could lead to effect estimates that do not exist or are in the opposite direction of the effects at the individual-level. Thus, when individual-level data are unavailable, this approach does not allow for conclusions about individual-level treatment effects. Combined with the biased outcome measure due to possibly missing data at certain hospitals [11], this warrants caution in interpreting treatment effects derived from this type of analysis [45]. In general, the ecological analysis would only be superior to individual-level analysis when the influence of hospital practice on treatment is independent of prognostic factors and the number of observations is sufficiently high.

Instrumental variable analysis has become increasingly popular in epidemiology as it can exploit the advantages of both individual-level and hospital-level approaches and can provide unbiased treatment effect estimates [46–48]. This is underscored by the fact that for IVT, our instrumental variable analysis results are in line with recent RCTs; among patients with acute large vessel occlusion stroke, these trials showed noninferiority of combined IVT and EVT relative to EVT alone regarding good functional outcome [18, 24, 25]. This was in contrast to other RCTs that failed to demonstrate noninferiority [22, 41]. In this analysis, the assumption of endogeneity due to unobserved confounding was violated, but still, in comparison to RCTs, instrumental variable analysis is superior to the conventional regression method. In addition, the results of previous RCTs have not been conclusive on the impact of the type of anesthesia on neurological outcome after EVT for stroke patients [20, 21, 49, 50]. However, comparing our results with those RCTs might not be valid since the comparator was different [51]. No violations of the instrumental variable assumption were noticed in the GA intervention analysis. This demonstrates that our instrumental variable analysis for this intervention was more efficient than for the IVT intervention and its results might be more valid than those of the conventional approaches. In instrumental variable analysis, a reliable instrumental variable must satisfy criteria related to the instrument and sample size to allow for adequate estimation of the treatment effect [52]. A comparison of the precision of the estimates was assessed by looking at the widths of the confidence intervals around effect estimates. Larger widths are interpreted as a less precise effect estimate. Weak instruments might produce large confidence intervals which result in imprecise (and biased) results. For the GA intervention, the intervals around the estimates were narrower using the instrumental variable approach than conventional approaches. For the IVT intervention, the intervals were broader, as a result of the weak instrument. For the IVT intervention, using hospital as an instrument might not be valid, since the receiving IVT is not very hospital dependent but more related to (unmeasured) contraindications at the patient level. In addition, the instrument was associated with some measured confounders. The adjusted instrument, by definition, is independent of those factors. On the other hand, the fact that we have measured confounders that predict the instrument makes it likely that other unmeasured predictors also predict treatment, that we cannot account for. Therefore, for reliability and validity of the instrumental variable analysis, it is essential that a suitable instrument is used to overcome unmeasured confounding. Reliability is mentioned as the most important limitation of instrumental variable analysis. Boef et al. [53] states that the statistical power of instrumental variable analysis is dependent on instrument strength and the strength of unmeasured confounding, but will usually be large given the typical moderate instrument strength in medical research. However, the increasing availability of data (sources) and existence of large registry databases, can accommodate the lack of statistical power of this method.

In summary, we recommend that investigators analyzing observational studies consider the sources of (un) measured confounding that may affect their results. The standard analysis should not be blindly accepted before considering what type of (un) measured confounding may contribute to the results. Several approaches can be used in different phases of study to account for unmeasured confounding [4]. Instrumental variable analysis might be used in conjunction with other approaches, such as multivariate regression analysis, propensity score methods, or ecological analysis to better describe the effect of treatment.

### Strengths and limitations

A major strength of our study is that we used the prospective data from a nationwide registry of all 17 EVT hospitals with various relevant case-mix variables which enable us to compare four approaches and demonstrate the influence of the analytical approach on the estimated effect.

The first limitation of the study is that we were not able to compare our treatment effect estimates to an estimate derived from an RCT with the same comparator. As a result, we cannot prove one approach as being superior to the others given our observational data. The second limitation might be that we only analyzed one clinical dichotomous outcome and no other outcomes with possibly other scales (i.e., ordinal, continuous). Thus, it is uncertain whether the results of this study are also generalizable to other outcomes. The third limitation is that we only used three approaches to compare the treatment effect. There are other approaches with probability of different results like the propensity score calibration and two-stage calibration statistical methods  [54–56] which can be an alternative approach for instrumental variable analysis; or propensity score matching and inverse probability of treatment weighting [42, 43] as an alternative to covariate adjustment. The fourth potential limitation is that for some of the models we compared (e.g. GEE), we made specific analytical choices that may have affected (the statistical significance of) our effect estimates. For example, in the GEE model, we assumed a compound symmetry correlation structure. While this is a common assumption in this type of research [57], it may not (fully) hold. Finally, it should be recognized that we deliberately included a limited number of covariates to keep consistency over different models, while adjusting for other patient or hospital characteristics, e.g., the time from arrival at the emergency department to EVT could have led to improved model performance and different treatment effect estimates.

## Conclusion

Using observational data, the choice of statistical approach has important consequences for the size, direction, and statistical significance of estimated treatment effects. Each approach has its merits and limitations and deals differently with the potential influence of (un) measured confounding. Before applying an approach and interpreting the results, researchers should understand these issues in relation to the specific characteristics of their data. While it is not desirable to appoint one approach as being always preferable, the instrumental variable analysis should be considered when unobserved confounding and hospital practice variation are expected in observational multicenter studies.

## Supplementary Information

**Additional file 1.**

## Data Availability

The data that support the findings of this study are available from the MR CLEAN Registry executive committee but restrictions apply to the availability of these data, which were used under license for the current study, and so are not publicly available. Data are however available from the authors upon reasonable request and with permission of the MR CLEAN Registry executive committee.

## Abbreviations

ED: Emergency department
GA: General anesthesia
GEE: Generalized estimating equation
IVT: Intravenous thrombolysis
mRS: Modified Rankin Scale
NIHSS: National Institute of Health Stroke Scale
