# Supplementary material for: Estimation of treatment effects in observational stroke care data: comparison of statistical approaches
Source: BMC Med Res Methodol. 2022 Apr 10;22:103. doi: 10.1186/s12874-022-01590-0 (PMC8996562; doi:10.1186/s12874-022-01590-0)
Supplement: Supplementary file 1 — Additional file 1. [file 12874_2022_1590_MOESM1_ESM.pdf]

SUPPLEMENTARY INFORMATION FOR:

ESTIMATION OF TREATMENT EFFECTS IN OBSERVATIONAL STROKE CARE DATA: COMPARISON OF STATISTICAL APPROACHES

Commands of *ivregress* in STATA

```
ivregress gmm good_outcome (ivtrom = ivtpercentage), wmatrix(cluster cnonr)
eform(OR)
estat endog
estat firststage

ivregress gmm good_outcome sex age prev_str prev_af prev_ht prev_hc NIHSS_BL
onset_to_er_every30min (ivtrom = ivtpercentage), wmatrix(cluster cnonr) eform(OR)
estat endog
estat firststage


ivregress gmm good_outcome (GA = GApercentage), wmatrix(cluster cnonr) eform(OR)
estat endog
estat firststage

ivregress gmm good_outcome sex age prev_str prev_af prev_ht prev_hc NIHSS_BL
onset_to_er_every30min (GA = GApercentage), wmatrix(cluster cnonr) eform(OR)
estat endog
estat firststage
```

Unadjusted	OR (95% CI): 1.58 (1.33-1.88); *P*<0.001
Case-mix adjusted[#]	OR (95% CI): 1.30 (1.04-1.62); *P*<0.001

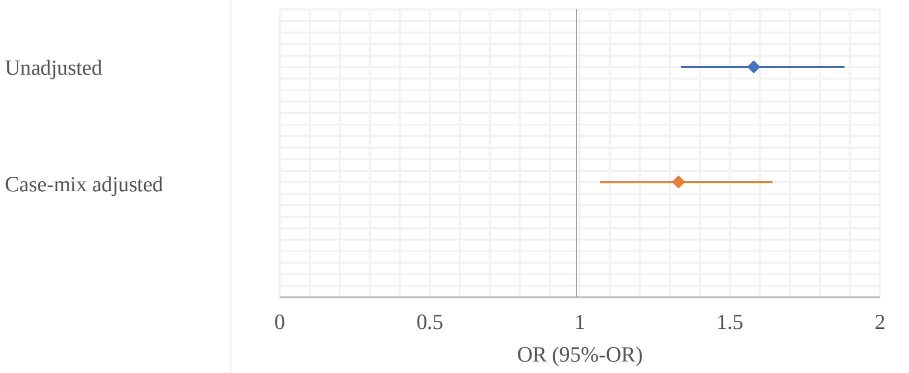

Supplementary Figure 1 Effect estimates of receiving IVT intervention on good functional outcome (mRS 0-2 at 90 days) from the generalized linear mixed model (GLMM) statistical method.

[#] Case-mix variables in the models are including age, sex, medical history, NIHSS score baseline, and time from onset to arrival at the ED of intervention hospital.

Unadjusted	OR (95% CI): 0.66 (0.50-0.87); *P*<0.001
Case-mix adjusted[#]	OR (95% CI): 0.60 (0.43-0.81); *P*<0.001

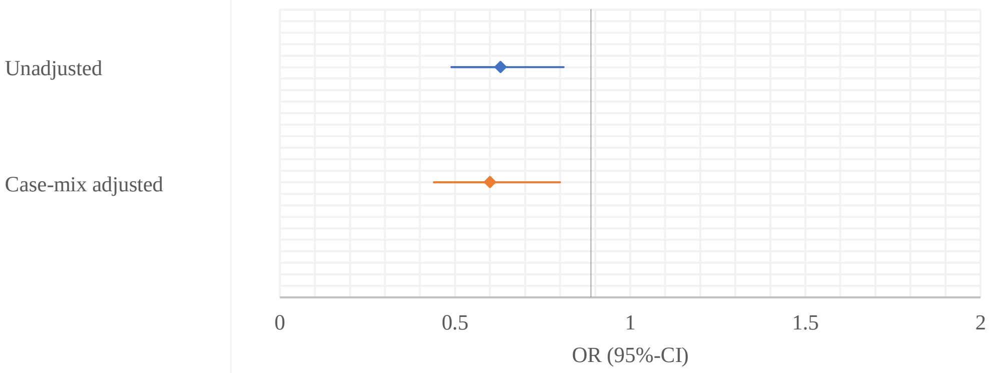

Supplementary Figure 2 Effect estimates of receiving GA intervention on good functional outcome (mRS 0-2 at 90 days) from the generalized linear mixed model (GLMM) statistical method.

[#] Case-mix variables in the models are including age, sex, medical history, NIHSS score baseline, and time from onset to arrival at the ED of intervention hospital.

Supplementary Table 1 Associations of case-mix variables with receiving IVT and the instrumental variable

	Receiving IVT		Instrumental variable*	
	OR (95% CI)	*P*-value	β (95% CI)	*P*-value
Age (years)	1.00 (0.99-1.00)	0.63	0.02 (0.01-0.04)	0.005
Men	1.10 (0.92-1.32)	0.31	0.11 (-0.31-052)	0.62
Medical History				
Previous Stroke	0.53 (0.42-0.66)	<0.001	-0.92 (-1.48- -0.35)	0.002
Atrial Fibrillation	1.10 (0.85-1.27)	0.70	-0.63 (-1.13- -0.12)	0.02
Hypertension	0.25 (0.12-0.31)	<0.001	-0.05 (-0.51-0.40)	0.82
Hypercholesterolemia	0.99 (0.70-1.07)	0.17	-0.24 (-0.72- 0.24)	0.33
Baseline NIHSS score	0.99 (0.98-1.00)	0.49	-0.07 (-0.10- -0.035)	<0.001
Time from onset to arrival at the ED (min)	0.92 (0.94-0.94)	<0.001	-0.01 (-0.06-0.03)	0.60

* Instrumental variable is intervention preference and defined as proportion of patients who received IVT within each hospital.

Supplementary Table 2 Associations of case-mix variables with undergoing GA and the instrumental variable

	Undergoing GA		Instrumental variable*	
	OR (95% CI)	*P*-value	β (95% CI)	*P*-value
Age (years)	0.99 (0.98-1.00)	0.06	0.03 (-0.07-0.12)	0.56
Men	1.10 (0.92-1.31)	0.30	1.31 (-1.17-3.81)	0.30
Medical History				
Previous Stroke	0.98 (0.76-1.25)	0.85	-1.75 (-5.15-1.65)	0.31
Atrial Fibrillation	0.86 (0.69-1.07)	0.18	-3.04 (-6.05- -0.04)	0.05
Hypertension	0.77 (0.64-0.94)	0.009	-3.31 (-6.04- -0.57)	0.02
Hypercholesterolemia	0.83 (0.67-1.02)	0.08	-2.54 (-5.41-0.33)	0.08
Baseline NIHSS score	1.02 (1.01-1.04)	0.002	-0.21 (-0.41- -0.01)	0.05
Time from onset to arrival at the ED (min)	0.99 (0.98-1.02)	0.91	0.16 (-0.12-0.44)	0.26

* Instrumental variable is intervention preference and defined as proportion of patients who underwent GA within each hospital.
